# The Use of Virtual Environments for Survey Spatial Ability Evaluation in Topographical Disorientation

**DOI:** 10.1155/2008/953640

**Published:** 2008-04-11

**Authors:** Francesca Morganti, Maria Luisa Rusconi, Anna Paladino, Giuliano Geminiani, Antonella Carassa

**Affiliations:** ^1^Applied Technology for Neuro-Psychology LabIstituto Auxologico ItalianoMilanoItaly; ^2^Institute of Psychology and Sociology of CommunicationUniversity of LuganoLuganoSwitzerland; ^3^Department of Human ScienceUniversity of BergamoBergamoItaly; ^4^Department of Neurological ScienceUniversity of MilanMilanItaly; ^5^Department of PsychologyUniversity of TorinoTurinItaly

**Keywords:** Spatial cognition, topographical disorientation, virtual reality, planning in advance

## Abstract

Due to their interactivity and to the sense of presence they afford, virtual environments constitute an interesting opportunity to study spatial cognition. In accordance with this perspective, we aimed to introduce a spatial test in virtual simulation in order to investigate the survey spatial ability in patients with topographical disorientation. To do this, we used the “planning in advance task” in a virtual environment that constitutes an effective procedure to experimentally evaluate survey maps. With this procedure we present the single case of a woman, with a right medial temporal lobe lesion, who shows a selective impairment in the acquisition of new spatial relationships. The patient’s performance in “planning in advance task” was compared with that of a control group made up of 40 female subjects matched for age and education. Results show how the patient revealed a significantly lower spatial performance when compared to the control group, demonstrating an inability to solve survey-type spatial tasks in complex virtual environments.

## 1. Introduction

Due to their interactivity and to the sense of presence they afford, the growth of virtual reality (VR) technologies has introduced new interesting ways to study spatial cognition and topographical disorientation [9,11]. The subjective involvement in such highly interactive systems supports people in "ecologically like" active environment exploration. This interaction allows people to organize spatial knowledge into route and survey maps in a way generally comparable to the learning obtained through the navigation of natural environments [12].

In literature, different types of inability to navigate through familiar environments or to learn novel routes in patients affected by topographical disorientation have been described.

Topographical disorientation has been associated with lesions due to stroke, traumatic injury or surgical treatment for epilepsy. The brain regions classically involved in the different varieties of TD are the right parietal lobe, the dorsal occipital parietal pathway and the medial temporal lobes [1,2,7].

In accordance with this perspective we aimed to introduce a spatial cognition evaluation using virtual simulations in order to investigate the survey spatial ability in patients with topographical disorientation. Survey maps are, in fact, flexible high level representations that

ISSN 0953-4180/08/$17.00 © 2008 – IOS Press and the authors. All rights reserved

Table 1
Neuropsychological evaluation. (*) indicates pathological score

| Test | Score |
|---|---|
| Coloured Raven P.M. 47 | 32/36 |
| Tower of London | 25/36 |
| Trial Making Test | |
| A | 53 |
| B | 154 |
| B-A | 81 |
| Token test | 32/36 |
| Boston Naming Test | 54/60 |
| Digit Span | 6.25 |
| Short Story Recall | 2.50* |
| Rey's words Recall | Immediate: 28.5, Delayed: 0* |
| Rey-Osterrieth Figure Recall | 1.6* |
| Rey-Osterrieth Figure Drawing | average |
| Corsi's Span | 4.50 |
| Learning a Spatial Sequence | 7.75 |
| Maze test | Deficit* |
| Road map test | 32/32 |
| Buildings Matching | 16/20 |
| Buildings Sorting | 20/20 |
| Popular Places Recognition | 24/26 |
| "What and Where" Test | Deficit* |
| Benton's Line Orientation Test | 26/30 |
| Street's Completion Test | 2.50 |

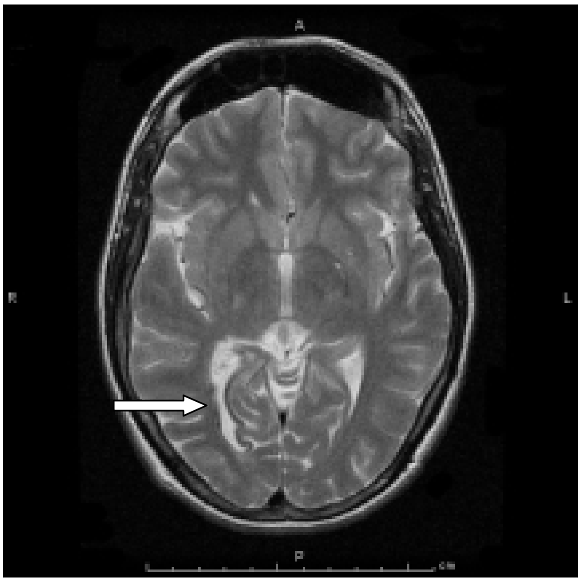

Fig. 1. MRI scan showing an haemorrhagic lesion in the right ventricular trigonal region with enlargement of the temporal ventricular horn.

provide agents with the possibility to infer and plan paths even in an unknown environment [4–6]. The main aim of our study is to investigate where impairment in survey map organization could be the key element for topographical disorientation manifestations in brain damaged subjects. In doing this we use the "planning in advance task" in a virtual environment, which constitutes an effective procedure to experimentally evaluate survey maps [10]. With this procedure, we tested the single case of a woman who suffered from an haemorrhagic lesion to the right medial temporal lobe.

## 2. Materials and method

### 2.1. Case history

R.G. is a 32-year-old, right-handed woman who suffered from an haemorrhagic lesion to the right medial temporal lobe. Two years after being discharged from hospital she revealed a persistent spatial orientation deficit: an impairment to learn environmental landmarks associated with an inability to recall the location of different spatial places. Her spatial abilities deficit doesn't interfere with everyday activities such as driving and finding her way in familiar locations.

At the time of testing with the use of virtual environments, she reported a selective impairment of the ability to find her way in new environments, whereas her performance in the cognitive tests was within normal range except for a deficit with reference to some verbal and visuo-spatial long-term memory tests.

The MRI scan, depicted in Fig. 1, shows a previous haemorrhagic lesion in the right ventricular trigonal region with an enlargement of the temporal ventricular horn (right temporo-occipital area and hippocampus).

### 2.2. Neuropsychological evaluation

The neuropsychological evaluation, summarized in Table 1, revealed impairment in two tests of verbal long-term memory, in one visuo-spatial long-term memory task and a selective deficit in the acquisition of new spatial relationships. In addition to the standardized neuropsychological evaluation phase, we introduced a situated assessment approach in order to highlight the patient's everyday difficulties in managing known and unknown environments. Where normative data was not available, a control group was included (twenty normal subjects, matched for age and schooling).

In order to evaluate the patient's ability to actively explore complex environments and to organize it into survey maps, a different evaluation methodology, based on virtual reality, was introduced.

Table 2
Patient performance in VR-based evaluation compared with a control group

| | Age | | Schooling | | Score | | | | |
|---|---|---|---|---|---|---|---|---|---|
| | Mean | SD | Mean | SD | Planning in advance (range 0–9) | Pointing (angular error) | Wayfinding (range 0–4) | Sketch-map accuracy | Sketch-map hierarchy |
| R.G. | 32 | | 18 | | 1 | unable to perform | 0 | Low performance | Low performance |
| Controls (40 subjects) | 26.27 | 1.98 | 16.5 | 1.5 | M = 4.52; SD = 2.31 | M = 67.14 SD = 16.47 | M = 3.3 SD = 1.05 | 14/40 (number of accurate maps/total of sketched maps) | 23/40 (number of hierarchical maps/total of sketched maps) |

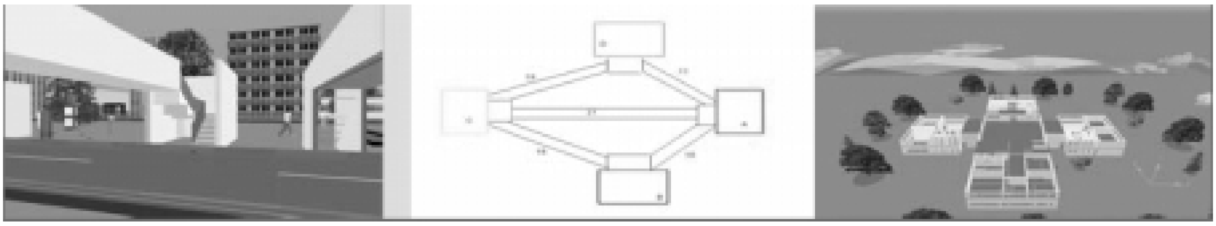

Fig. 2. The three environments employed in the study. From left to right: a snapshot of the training environment, the plan of the planning in advance environment and a snapshot of the survey phase environment.

### 2.3. *Virtual reality evaluation*

In accordance with a previous experimental work in the same research field [10], three different virtual environments were employed in this study.

The first environment is a small one, used for training purposes. The second environment was especially designed to assess the ability of "planning in advance" optimal paths. The third one well supports the performance evaluation in three survey tasks (wayfinding, pointing, and sketch map), as described in a previous experimental study [3].

Figure 2 shows snapshots and a plan of the three environments.

All virtual environments were run on a PC Pentium II, 400 MHz and explored in an immersive way by a virtual reality Head Mounted Display with 60° horizontal angle. The translation on the horizontal axis within environments was controlled by a joystick, where the rotation was performed by a rotating head via the Inter-Trax Intersense gyroscopic sensor.

The VR-based evaluation consisted of a first training phase in which the patient had the opportunity to learn how to navigate the first virtual environment through immersive interaction. This environment has the same architectural and interactive features of the virtual environments employed in the assessment phase.

A "planning in advance" phase followed, in which the patient was evaluated on route planning or navigation task performance. She was firstly requested to explore the second environment by navigating into it. Then, starting from a fixed point, she was requested to reach different target places avoiding unexpected obstacles presented during the task execution.

Finally, the patient was involved in a typical survey assessment phase in which she was requested to explore the third environment and to perform pointing, wayfinding and sketch map tasks, generally considered as survey-type spatial tasks.

In line with Morganti et al.'s [10] procedure, path plan performance in the planning in advance evaluation were analysed and evaluated on a 0–9 range. For the pointing task, the angular error between pointing direction and target point was calculated. To evaluate wayfinding, a 0–4 range score was assigned to the ability to perform optimal wayfinding by creating shortcuts between different places. For the sketch map task, three independent judges evaluated the presence of accuracy and the cluster organization (hierarchy) in the map drawings.

Spatial ability in the "planning in advance" phase has been correlated with performance on survey spatial tasks provided in the survey assessment phase.

## 3. Results

The patient's performance in VR-based evaluation was compared with that of a control group made up of

40 female subjects matched for age and education (Average age: 26.27 years old, SD: 1.98; Average school leaving age: 16.5, SD: 1.5). Results are showed in Table 2.

Virtual reality-based planning in advance task showed the patient's inability to find her way in unknown environments, not referable to landmark recognition and memorization, the characteristics of which are correctly detected but not spatially located. When compared with the control group the patient presented a survey-type performance significantly different from the average (Planning in advance z score $= 0.95$; Pointing z score $= -4.07$; Wayfinding z score $= -3.14$).

In particular, virtual reality-based evaluation highlighted in our patient a selective impairment to infer relationships among distant landmarks, to plan shortcuts in order to find a target point and to revise path planning in order to avoid unexpected obstacles. Finally, the deficits observed in planning tasks appear to be correlated with accuracy and hierarchical organization in the sketch map task of the third large scale environment.

## 4. Conclusions

Planning in advance performance highlights how the patient's ability to plan a path in advance and to modify it in order to avoid obstacles appears to be significantly lower when compared to the control group performance in the same environment. The ability to plan new paths in advance and to refine them in order to avoid unexpected obstacles can be considered a distinctive feature of a survey-type spatial organization [10].

Pointing, wayfinding and sketch-map tasks requested in the third VR environment confirmed an inability to organize spatial knowledge into a survey map. Our patient seemed to be able to recognize salient places but revealed an impairment to estimate a target place direction (pointing), to use landmark places during the exploration of the environment in order to reach a target place (wayfinding) and to infer relationships among distant places previously explored (sketch-map).

Although the precise role of different neuroanatomical areas involved in route/survey maps remains controversial, the impairment resulting from VR tasks could be compatible with the lesion observed in our patient [8, 13].

Finally, VR-based evaluation provides the possibility to highlight in some patients a spatial impairment in everyday situations. A VR-supported evaluation approach, in fact, allows an immediate and direct interaction that is generally possible in daily environments and constitutes an important vehicle through which spatial ability can be assessed. It also provides the possibility to have an egocentric point of view during interaction that will highlight a patient's difficulty in the translation of survey spatial knowledge, derived for example from a paper and pencil maze test, into a route one.

Moreover, the VR-based evaluation used in our study, due to the situated nature, doesn't require memory efforts that appear to be impaired in our patient. The patient, in fact, during the evaluation was repeatedly requested to actively explore the environments and to solve spatial tasks that don't require a retention of verbal information. For this reason, we can reasonably conclude that an impairment in Short Story Recall and Rey's Words Recall tests have not interfered with spatial tasks proposed in virtual environments.

Our VR evaluation provided us the possibility to assess the patient's impairment in coping with unknown environments by analyzing her active exploration and it can be considered as a more situated approach than the assessment generally used in laboratory settings. Accordingly, we can conclude that the integration of virtual reality with traditional neuropsychological evaluation may contribute to an improvement in the classical paper and pencil-based evaluation of topographical disorientation. In particular, with the introduction of low cost virtual reality systems and the booming market of 3D environments (generally developed for gaming purposes), the use of VR doesn't require sophisticated equipments and constitutes, at the present time, a great opportunity for neuropsychological research studies.
